# Quality assessment and control of high-throughput sequencing data

**DOI:** 10.3389/fgene.2014.00235

**Published:** 2014-07-25

**Authors:** Mick Watson

**Affiliations:** Edinburgh Genomics, The Roslin InstituteEdinburgh, UK

**Keywords:** bioinformatics, genomics, transcriptomics, quality control, next-generation sequencing, sequencing, illumina

Second- and third-generation sequencing technologies are driving a revolution in biology and medicine, with ultra-high throughput sequencers now able to produce 200 human genomes every 3 days at a cost of $1000 per genome (Watson, 2014). Meanwhile, in our lab at Edinburgh Genomics and in other labs throughout the World, researchers are generating their first single-molecule reads from a hand-held, USB-powered sequencer as part of Oxford Nanopore's MinION access programme (MinION Access Programme, 2014^1^). Whilst the revolution in biology is recognized, the associated revolution in bioinformatics often goes unmentioned. This Frontiers “Research Topic” is about that revolution; it is about data, and data-driven discovery. The sequencers mentioned above, and others from Pacific Biosciences and Ion Torrent, produce either huge amounts of data, data that are very complex, or both. Bioinformaticians throughout the world are creating novel pipelines, algorithms and tools to be able to cope with the huge amount of diverse data types that can be produced. The very first step in many of those pipelines and tools is quality assessment, quality control and artifact removal. These issues all involve data-driven research—what can we learn from the data? What are the data telling us about quality and artifacts? The first group of papers in the research topic deal with quality assessment and reveal pipelines that are in use in sequencing facilities today. The second set of papers deal with applications of sequencing technologies to particular domains, and how we can improve those applications through effective control of quality and artifacts. The final set of papers deal with very specific biological questions, and what we can learn from the raw data to improve our analyses and help us to better answer those questions.

A series of bioinformatics pipelines are applied to sequencing data by the data generating facility, and it is important that those who work with sequencing data understand these. Leggett et al. (2013) reveal many of the pipelines and tools used at The Genome Analysis Centre (TGAC), a genomics institute based in Norwich, UK, which has access to every major sequencing platform. Their paper describes every step in the data generation pipeline, from their Laboratory Information Management System (LIMS) to data-specific pipelines for mate-pair and RAD-Seq libraries. Similarly, Paszkiewicz et al. (2014) describe bioinformatics quality-control pipelines used at the Exeter sequencing service, a smaller laboratory at the University of Exeter. Interestingly, they also include details of laboratory quality-control where relevant. Hadfield and Eldridge (2014) describe MGA, a tool that deals with two of the most frequently asked questions in DNA sequencing facilities: “how much did we sequence?” and “what did we sequence?” MGA presents a visual and tabular report to users detailing the yield for each lane in a sequencing run, as well as an assessment of the amount of adapter and contaminant DNA present. Finally, Trivedi et al. (2014) describe an important pipeline/method in use at Edinburgh Genomics, a genomics facility at the University of Edinburgh. Many QC metrics involve mapping to a reference genome, and Trivedi et al ask a simple question: what can we learn when there is no reference genome? Their paper deals with assessment of insert size, duplication rate and contamination by first generating a simple and fast *de novo* assembly of the data, and they show that even when the assembly is poor, accurate QC metrics can be generated.

The second group of papers deals with the application of quality control to specific types of sequencing library. Macmanes (2014) looks at the effect of quality trimming on mRNA sequencing data, and specifically assembly of those data. Quality trimming is ubiquitous in many bioinformatics pipelines, with many choosing an arbitrary Q-score of 20 or 30 as a cut-off. However, MacManes shows that such “aggressive” trimming can have a large effect on the results, and suggests a much lower Q-score cut-off if the purpose of the experiment is transcript discovery. Mbandi et al. (2014) tackle exactly the same problem, and come to similar conclusions, showing that transcript assemblies from untrimmed data result in better alignments and more contiguous assemblies. Finally, Turner (2014) describes a bioinformatics protocol for assessing insert size and adapter content in Nextera XT libraries. The Nextera XT protocol uses transposomes to fragment DNA and is very sensitive to the concentration of input DNA. These libraries can result in fragments that are far smaller than the read length, and Turner describes a protocol that can cope with this problem.

The final group of papers deal with quality and artifact assessment applied to specific biological problems. Amaral et al. (2014) describe the problem of RNA-Seq in small invertebrates. As the traditional RNA-Seq protocol requires a relatively large amount of RNA as input, invertebrates such as *Drosophila* need to be pooled prior to sequencing. This pooling can produce additional complexity in the data, and Amaral et al. describe the use of marker genes to identify data points that suffer from contamination and should be removed from further analysis. Complementary to Macmanes (2014), Mbandi et al. (2014) and Amaral et al. (2014) find that trimming of the 5′ end, to remove biases introduced by random hexamer priming (Hansen et al., 2010), improves the mapping rate. Carroll et al. (2014) deal with ChIP-Seq and ChIP-exo data and suggest that standard metrics for assessing the quality of these data types should be assessed at every step of data pre-processing. They also suggest that ChIP-exo data requires different processing steps and an adaptation of cross-correlation metrics. Finally, Kumar et al. (2013) describe a method for discovery and removal of “contaminants,” but here the term is extended to include symbionts and parasites. They show that “Blobology,” separation of assembled contigs using GC content and coverage, can be used to bin data into distinct groups that can then be assembled separately, resulting in better assemblies and useful data from organisms that may be difficult to study in isolation.

The papers in this Frontiers Research Topic represent a diverse and fascinating collection, and should be of interest to a wide audience, including bioinformaticians keen to improve their pipelines and biologists keen to learn more about the complexity of second- and third-generation sequencing data.

## Conflict of interest statement

The author declares that the research was conducted in the absence of any commercial or financial relationships that could be construed as a potential conflict of interest.

## Footnotes

^1^https://www.nanoporetech.com/technology/the-minion-device-a-miniaturised-sensing-system/minion-access-programme
